# Development and validation of a dual-perspective competency scale for novice medical laboratory scientists in Japan

**DOI:** 10.1186/s12909-026-08626-9

**Published:** 2026-01-19

**Authors:** Kiriko Maekawa, Daisuke Sugiyama, Takamitsu Imanishi, Tsumugi Oki, Kenichiro Ohnuma, Mary O. Carayannopoulos, Seimi Satomi-Kobayashi, Seiji Kawano

**Affiliations:** 1 Department of Community Medicine and Social Healthcare Science, Division of Medical Education, Kobe University Graduate School of Medicine, 2-1-5 Aratacho, Hyogoku, Kobe, Hyogo 652-0032 Japan; 2 Faculty of Nursing and Medical Care, Keio University, 4411, Endo, Fujisawa, Kanagawa, 252-0883 Japan; 3 Department of Clinical Laboratory, Kobe University Hospital, 7-5-1 Kusunokicho, Chuoku, Kobe, Hyogo 650-0017 Japan; 4 Department of Pathology, Rutgers Robert Wood Johnson Medical School, New Brunswick, NJ 08901 USA; 5 Department of Internal Medicine/Rheumatology, Asago Medical Centre, 392 Hokkoji, Wadayamacho, Asago, Hyogo 6695267 Japan

**Keywords:** Confirmatory factor analysis, Competency, Exploratory factor analysis, Instrument, Medical laboratory scientists, Scale, Validity

## Abstract

**Background:**

Competency-based education defines competencies as educational outcomes and guiding instruction and assessment. In Japan, standardised competency frameworks for medical laboratory scientists in clinical settings are underdeveloped. This study aimed to develop and validate a competency scale for novice medical laboratory scientists with less than 3 years of clinical experience into an assessment tool.

**Methods:**

This study involved scale development and validation conducted in four phases: a preliminary study to test content validity for exploratory purposes, a pilot survey to examine the feasibility of the evaluation sheet under conditions similar to those of the main survey, a main survey to verify reliability and validity and a final phase to establish the completed scale. In the main survey, each novice medical and biomedical laboratory scientist (self-evaluator) was paired with two supervising evaluators from the same institution. The supervising evaluators, comprising educational supervisors, senior medical laboratory scientists or managers, were directly involved in the guidance and assessment of the corresponding novice.

**Results:**

Exploratory factor analysis of the self-evaluation dataset identified four factors with 51 items using a five-point Likert scale. The cumulative variance explained by the four factors was 52%, and Cronbach’s alpha ranged from 0.78 to 0.94 for each factor. The validity of the scale was confirmed through confirmatory factor analysis with the supervisory evaluation dataset.

**Conclusions:**

The Japan Medical Laboratory Scientists Competency Scale demonstrated robust psychometric properties, establishing it as an effective tool for assessing novice medical laboratory scientists in Japan through self- and supervisory evaluation. Those findings would support enhancements in workforce development and competency-based education in this field.

**Supplementary Information:**

The online version contains supplementary material available at 10.1186/s12909-026-08626-9.

## Background

Competency-based education (CBE) is defined as ‘an outcome-based approach to education that incorporates modes of instructional delivery and assessment efforts designed to evaluate mastery of learning’ [1]. Competencies serve as the foundation for designing educational programmes and evaluating their achievements. In medical education, Frenk et al. [2] emphasised the design of competency-based frameworks flexible enough to accommodate diverse clinical roles across interdependent systems. For example, the Accreditation Council for Graduate Medical Education (ACGME) framework [3] comprehensively captures professional abilities and informs programme design and assessment. CBE has been adopted beyond physician training, extending to other healthcare professions [4–7], increasing the need for developing evaluation methods and assessment scales to align with defined competencies [8–10].

As CBE has expanded, standardised competency assessment instruments have been developed across various health professions [11, 12]. These tools evaluate the extent to which learners demonstrate profession-specific competencies, thereby supporting educational design and outcome evaluation. In developing such instruments, many studies have employed factor analysis to identify underlying competency subscales and ensure construct validity [13–15]. This approach is particularly valuable because factor analysis reveals latent structures that are not directly observable, facilitates the selection of items that strongly represent intended constructs and confirms whether the factor structure aligns with theoretical expectations [16, 17].

In the profession of medical (biomedical) laboratory scientist (MLS), the International Federation of Biomedical Laboratory Science (IFBLS) [18] and the Centres for Disease Control and Prevention [19] have defined competencies for biomedical laboratory scientists and public health laboratory professionals. In the United States, the American Society for Clinical Pathology provides competency standards for clinical laboratory education [20]. Professional titles for these roles vary internationally, such as medical laboratory scientist, biomedical laboratory scientist, medical technologist or clinical laboratory scientist. In this paper, the term ‘*medical (biomedical) laboratory scientist (MLS)*’ is used to encompass these designations collectively. Beyond international guidelines, recent research on MLS competencies [21, 22] has emerged, indicating growing interest in standardised training and assessment approaches. However, existing competency scales for the MLSs have not undergone sufficient validation through rigorous psychometric methods, such as factor analysis, and evidence of their reliability and validity remains limited.

In contrast to other countries, such as the United States, the MLS qualification system in Japan incorporates additional requirements, including physiological examinations and specimen collection [23]. Consequently, direct adoption of competency standards from other countries without adaptation is challenging and requires modifications that are suited to the Japanese clinical context. However, a standardised framework for assessing competencies among novice MLSs in Japanese clinical settings remains underdeveloped [24].

In educational evaluation, instructors employ assessment forms for summative and formative evaluations, whereas learners conduct self-assessments to reflect on their competency development [25]. A shared competency-based assessment form, which is used by both educators and learners, can be applied across varied educational settings and is considered effective for improving educational outcomes. In healthcare professions, instruments incorporating both self- and hetero-evaluations have been developed and validated [26, 27]. However, such dual-perspective competency assessment tools are lacking in the MLS field.

Against this background, as an initial step, we identified the minimum requirements for nationally licensed MLSs and defined them as competencies for novice MLSs. We then developed a list of competencies required for novice MLSs in Japan, as previously reported [24]. Building on this foundation, this study refined these competencies and transformed them into an assessment form completed by both novice MLSs and their educators. To the best of our knowledge, this is the first study to develop and validate a competency-based assessment tool for novice MLSs in Japan that enables self- and supervisory evaluations within the same framework. Here, we report analyses of its usefulness and the relationship between self- and supervisory assessments.

## Methods

### Study design

**Fig. 1:**
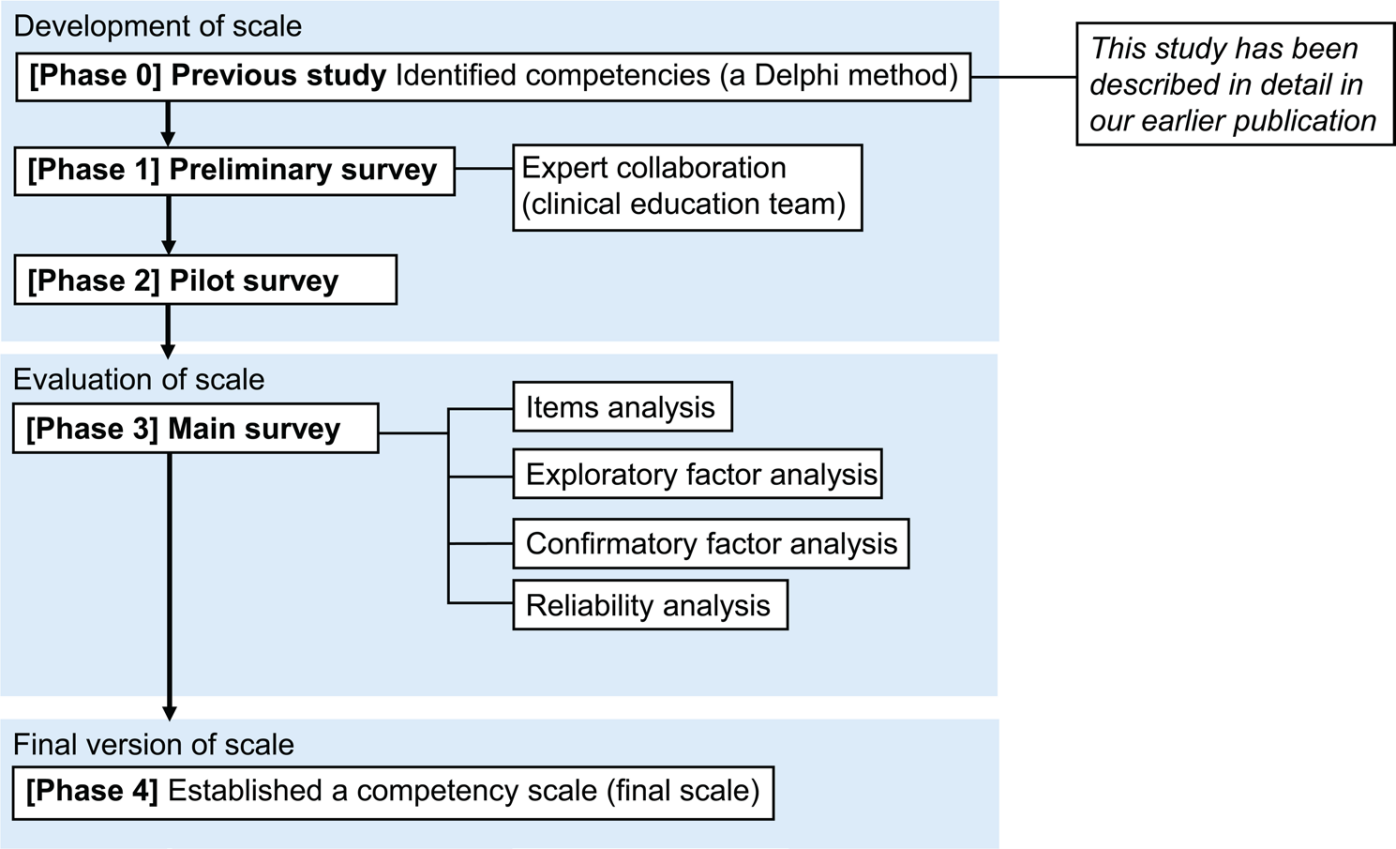
Development process of the competency scale. Overview of the four-phase process to develop and validate a competency scale for MLSs. Details of each phase are provided in the Methods section

This study aimed to develop a competency scale through a structured process. Item generation had been completed in a previous study (Phase 0); the present study comprised four subsequent phases. Phase 1 assessed the content validity of the initial evaluation sheet through a preliminary internal survey for exploratory purposes. Phase 2 examined the feasibility of the evaluation sheet under conditions similar to those of the main survey through a regional pilot survey. Phase 3 verified the validity and reliability through a nationwide survey as the primary data collection. Phase 4 finalised the competency scale. Phases 0–2 focused on scale development, Phase 3 on evaluation and Phase 4 on establishing the final version (Fig. 1). The design and procedures adhered to the COnsensus-based Standards for the selection of health Measurement INstruments (COSMIN) guidelines [28, 29] to ensure methodological rigour.

### Scale development

#### Phase 0: previous study

Competency items for Japanese novice MLSs were already revealed in our prior study [24]. This study focused on novice MLSs, defined as those with less than 3 years of post-graduation experience [30]. We employed a comprehensive literature review and a formal expert consensus process using the Delphi method [31] to generate and refine items. The response rates were 106/400 (26.5%) in Round 1 and 95/106 (89.6%) in Round 2. The participants’ mean professional experience (mean ± standard deviation [SD]), was 32.4 ± 6.0 years. Consensus rates exceeded 99.1% across categories, with 95 expert opinions ultimately converging. This strategy ensured robust content validity, as experts assessed item relevance, comprehensiveness and comprehensibility following COSMIN recommendations. Consequently, 54 competency items were identified as the minimum requirements and designated as core competencies for licensed novice MLSs. The items were organised into eight domains: Sample Collection, Preparation and Analysis (General), Preparation and Analysis (Specimens), Preparation and Analysis (Physiology), Medical Safety Management, Interpersonal Relationships, Research Ability and Ethics.

In this study, ‘*novice MLSs*’ were defined as individuals with less than 3 years of clinical experience as licensed MLSs, consistent with the definition in the prior study. The competencies reflected the unique professional responsibilities and expectations within the Japanese clinical setting.

#### Phase 1: preliminary survey

The preliminary survey assessed the content validity of the initial evaluation sheet, evaluate item clarity and response patterns and refine the sheet for the subsequent pilot survey [32].

To prepare for the survey, an initial evaluation sheet was developed based on the competencies identified in a prior study [24]. The preliminary survey was conducted using a questionnaire with an evaluation sheet, comprising 54 competencies and a five-point Likert scale, along with a free comment box.

Two rounds of internal surveys were conducted within the Department of Clinical Laboratory at Kobe University Hospital. Participants were recruited, comprising novice MLSs who served as self-evaluators and experienced MLSs who served as supervising evaluators. In this study, eligibility criteria were as follows: self-evaluators were licensed MLSs with less than 3 years of clinical experience who assessed their own competencies; supervising evaluators were licensed MLSs with more than 10 years of clinical experience [33, 34] who served as educational supervisors, senior MLSs or managers responsible for guiding and assessing the self-evaluating novice MLSs within the same institution.

The study’s purpose and procedures were explained to all MLSs in the Department of Clinical Laboratory at Kobe University Hospital to ensure understanding and obtain informed consent prior to questionnaire distribution. In both survey rounds, each novice MLS completed a self-assessment, while two supervising evaluators independently assessed the same individual. The first and second rounds involved distinct groups of self-evaluators. The first survey utilised paper-based questionnaires, whereas the second was conducted online.

Following each round, results were reviewed, focusing on item phrasing, the Likert scale and completion time. The questionnaire was refined through two rounds of surveys and expert discussions among the research team, including clinical education members (authors TI, TO and OK), who were MLSs in the hospital’s clinical laboratory department and involved in training novice MLSs. Their input informed the revisions. The total sample comprised 11 self-evaluators and 22 supervising evaluators. These refinements improved the evaluation sheet’s clarity and applicability.

These procedures followed COSMIN guidelines for assessing content validity [35], incorporating expert feedback to confirm that items were relevant, clear and comprehensive. Collaboration with the clinical education team members was integral, as their practical experience and contextual insights ensured the scale accurately reflected competencies required in clinical laboratory settings. Their feedback in each round directly enhanced item relevance and face validity, increasing the instrument’s applicability and credibility in clinical education.

#### Phase 2: pilot survey

The pilot survey was conducted under conditions similar to those of the main survey to examine item performance, comprehensibility, and feasibility of the revised evaluation sheet. Item clarity, response distributions, and completion time were evaluated, and final modifications were implemented prior to the main survey [32].

The survey utilised a questionnaire incorporating the refined evaluation sheet, developed through prior preliminary surveys. The questionnaire included revised, rephrased items assessed on a five-point Likert scale, demographic questions and a free-comment section.

Participants were recruited via email using a snowball sampling method. In addition, invitation letters were mailed to hospitals with more than 300 beds that were registered with the Ministry of Health, Labour and Welfare in Japan [36]. These hospitals were selected based on their capacity to employ sufficient MLSs and maintain structured educational systems. The targeted hospitals were located in western Japan. Both recruitment methods detailed the study’s purpose, significance and registration.

Eligibility criteria for evaluators remained consistent with Phase 1. Each self-evaluator was paired with two or three supervising evaluators. Questionnaires included a section confirming informed consent, completed by all participants. To ensure confidentiality, responses from self-evaluators and supervising evaluators were collected separately to prevent identification of individual answers.

Of the 127 hospitals invited to participate in the pilot survey, 36 (28%) agreed to participate. The sample comprised 43 self-evaluators and 93 supervising evaluators. No ceiling or floor effects were observed [37], and all absolute item scores fell within ± 1, indicating balanced response distributions without extreme skewness. Item-total correlation coefficients ranged from 0.47 to 0.82, confirming strong internal consistency and alignment of each item with the overall construct. Good–Poor analysis revealed statistically significant differences (*p* < 0.001) between the top and bottom quartiles for all items [38].

Based on the statistical analyses and qualitative feedback from participants, the evaluation sheet was further refined. These findings informed the preparation of the final evaluation sheet for the main survey.

## Scale evaluation

### Phase 3: main survey

The main survey aimed to evaluate the validity and reliability of the final evaluation sheet, established as a competency scale. Data were collected through a nationwide survey involving a substantial number of Japanese MLSs. Statistical analyses, including factor analysis, were conducted to assess the scale’s validity and reliability.

#### Questionnaire

The main survey utilised a questionnaire incorporating the final evaluation sheet, developed through the pilot survey. The questionnaire included a refined evaluation sheet with rephrased items assessed on a five-point Likert scale and demographic questions. Self-evaluators were asked about hospital size (number of beds and MLSs), years of experience as an MLS, experience in other occupations, age, gender and educational background. Supervising evaluators provided information on their position, gender, gender differences with the self-evaluator, duration in the same department, shared working hours and whether they directly trained the self-evaluator. Informed consent was obtained from all participants upon questionnaire completion, and responses were collected anonymously to ensure confidentiality.

#### Measurements

A competency scale was developed using a five-point Likert format with the following options: 1 = Poor, 2 = Needs Improvement, 3 = Acceptable, 4 = More than Acceptable, 5 = Outstanding and an additional Not Applicable (N/A) option. In Japan, MLSs rotate through various departments, such as Clinical Physiology and Specimen Testing, resulting in significant variation in their roles and responsibilities [23]. Consequently, some tasks or factors may be outside their experience. To address this, the N/A option was included for self-evaluators to mark items beyond their direct experience and for supervising evaluators to indicate items they had not personally observed.

Participation requests were sent via postal mail in sealed envelopes, while registration and survey responses were collected using Smooth Contact (WEBLIFE Inc., Japan), a platform enabling customised survey forms.

#### Participants

The main survey was conducted from February to April 2024, with all participants recruited via postal mail.

Eligibility criteria for hospitals mirrored those in Phase 2, requiring registration with Japan’s Ministry of Health, Labour and Welfare [36] and a minimum of 300 beds as of January 2024. However, Phase 3 expanded to a nationwide scope, excluding hospitals near the 2024 Noto Peninsula Earthquake-affected area. Of the 899 hospitals contacted, 101 participated.

Participant eligibility criteria were largely consistent with Phase 1, except that supervising evaluators were required to have at least 6 months of experience working with the corresponding self-evaluator, either currently or previously, within the same laboratory section. This 6-month period ensured sufficient observation for evaluations. Each participant unit comprised one self-evaluator and two supervising evaluators. Multiple submissions per hospital were permitted.

#### Statistical methods for the main survey

All statistical analyses were performed using R version 4.5.0 (R Foundation for Statistical Computing, Vienna, Austria). Item analysis evaluated ceiling and floor effects, item-total correlation coefficients and Good–Poor analysis. Exploratory factor analysis (EFA) was conducted on self-evaluator samples using maximum likelihood (ML) estimation with Promax rotation to examine the underlying item structure and assess construct validity, as this method accommodates correlated factors. A factor loading cut-off of 0.40 [39] was applied to identify items strongly associated with each factor, facilitating interpretation of the factor structure. The Kaiser–Meyer–Olkin (KMO) measure of sampling adequacy and Bartlett’s test of sphericity were used to assess data suitability for EFA. The KMO value of 0.90 indicated excellent sampling adequacy, and a significant Bartlett’s test confirmed that the correlation matrix was not an identity matrix, supporting EFA suitability [40, 41]. Moreover, internal consistency was evaluated using Cronbach’s alpha. Confirmatory factor analysis (CFA) was performed on supervising evaluator samples to examine generalisability of the factor structure identified through EFA. This enabled the structural validity of the model to be tested from a supervisory perspective, rather than relying solely on self-evaluation data.

The model was estimated using the ML method with the NLMINB optimisation algorithm. Only complete cases with no missing data were included in the analysis. Model fit was evaluated using multiple indices, including Comparative Fit Index (CFI), Tucker–Lewis Index (TLI), Root Mean Square Error of Approximation (RMSEA), and Standardised Root Mean Square Residual (SRMR). Acceptable model fit was defined as CFI and TLI values > 0.90 and RMSEA and SRMR values < 0.08 [42, 43].

Self-evaluators were categorised into three groups based on years of experience: less than 1 year (*first-year*), one to less than 2 years (*second-year)* and two to less than 3 years (*third-year*). Corresponding supervising evaluator scores were compared across these groups using linked identifiers, enabling paired analysis between self- and supervisory evaluations. Mean differences for each item were calculated to identify trends in competency scores by years of experience.

Items with a high proportion of N/A responses were considered to potentially affect reliability and consistency [44]. Although N/A responses differ from unanswered items, they are treated as missing data in statistical analyses, such as factor analysis and reliability testing [45, 46]. Items with N/A responses exceeding 10% of total responses may reduce item-level interpretability and psychometric integrity. Such items were excluded from further analyses.

#### Ethical considerations

This cross-sectional survey involved no interventions; thus, clinical trial registration was not required.

Ethics approval was obtained from the Institutional Review Board of Kobe University Graduate School of Medicine (approval number: B220149). All participants provided informed consent before completing the survey. To ensure anonymity, responses were collected and managed using unique identifiers, and data were used exclusively for research purposes.

### Final version of the scale

#### Phase 4

The competency scale was finalised utilising the COSMIN evaluation process. The scale’s title and factor structure were determined with input from MLS experts.

All scale items were originally developed and refined in Japanese through a Delphi process, a preliminary survey, and a pilot survey. Throughout these stages, expert reviewers confirmed that each item represented a single, distinct competency without redundancy or conceptual overlap.

During the translation of the finalised items into English, careful attention was paid to preserving the original meaning. However, due to linguistic and structural differences between Japanese and English, some nuances in the English wording could be interpreted as double-barreled or ambiguous. These issues were attributable to the translation process rather than to the original Japanese item construction. The English wording was therefore refined to ensure that each item clearly reflects a single, distinct concept.

## Results

### Samples and items for analysis

**Fig. 2:**
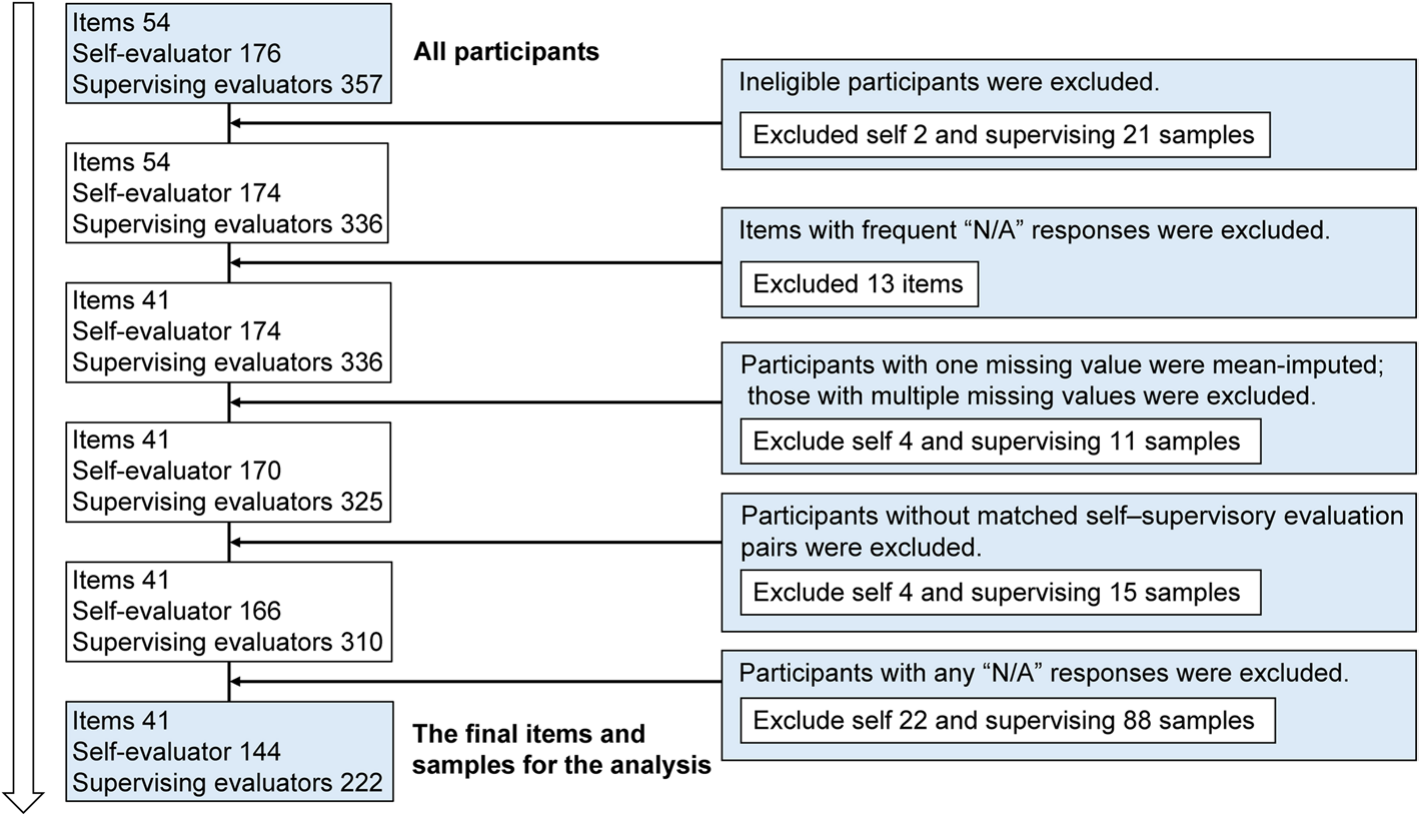
Sample selection process. Participants and items were screened for data quality. Ineligible participants and items with excessive N/A responses were excluded. Unmatched or missing self- and supervisory evaluations were also excluded. The final dataset comprised 41 items, 144 self-evaluations and 222 supervisory evaluations

To ensure data quality, five steps were applied before the analyses. First, ineligible participants were excluded. Second, items with frequent N/A responses were excluded. Items exceeding 10% N/A responses were concentrated in three domains influenced by institutional rotation systems: specifically, two from Sample Collection, six from Preparation and Analysis (Specimens) and five from Preparation and Analysis (Physiology). These constituted all items originally in those domains. Third, for participants with a single missing response, the value was imputed using the item’s mean score across participants [47, 48]; those with multiple missing responses were excluded. Fourth, only matched self- and supervisory evaluation pairs were retained. Finally, responses containing N/A answers were removed. The final dataset comprised 41 items, 144 self-evaluations and 222 supervisory evaluations (Fig. 2).

### Demographic characteristics of the samples

Of the 144 self-evaluators, most were affiliated with hospitals having 300–499 beds (*n* = 76, 52.8%), followed by 500–799 beds (*n* = 51, 35.4%) and 800 or more beds (*n* = 17, 11.8%) (Table 1). Regarding MLS staff numbers, the largest group consisted of 50 or more MLSs (*n* = 49, 34.0%), followed by 20–29 MLSs (*n* = 37, 25.7%), 30–39 MLSs (*n* = 32, 22.2%) and fewer than 20 MLSs (*n* = 6, 4.2%). Hospital size was determined by bed count, while laboratory scale was indicated by MLS staff numbers.

**Table 1:** Participants’ characteristics in the final samples

Characteristics of final samples			N	%
**Hospitals**			***N* = 144**	
Number of beds (Hospital Size)	1	300–499	76	52.78
	2	500–799	51	35.42
	3	≥ 800	17	11.81
Number of MLSs	1	< 20	6	4.17
	2	20–29	37	25.69
	3	30–39	32	22.22
	4	40–49	20	13.89
	5	≥ 50	49	34.03
**Self-evaluators**			***N*** = 144	
Experience as an MLS (months)	1	< 6	0	0
	2	≥ 6 and < 12	45	31.25
	3	≥ 12 and < 18	13	9.03
	4	≥ 18 and < 24	40	27.78
	5	≥ 24 and < 30	7	4.86
	6	≥ 30 and < 36	39	27.08
Prior work experience	1	Yes	4	2.78
	2	No	140	97.22
Age		Mean	24.17	-
		SD	1.45	-
		Missing	1	-
Gender	1	Male	44	30.56
	2	Female	99	68.75
		Missing	1	0.69
Highest education level	1	Diploma	26	18.06
	2	Associate degree	4	2.78
	3	Bachelor’s Degree	104	72.22
	4	Master’s Degree	9	6.25
	5	Doctoral Degree	0	0.00
		Missing	1	0.69
Evaluation time (mins)	1	< 15	122	84.72
	2	15–29	22	15.28
	3	30–44	0	0.00
	4	≥ 45	0	0.00
**Supervising evaluators**			***N*** = 222	
Experience as an MLS (years)	1	10–19	115	51.80
	2	20–29	62	27.93
	3	≥ 30	45	20.27
Holding a managerial position	1	Yes	163	73.42
	2	No	58	26.13
		Missing	1	0.45
Gender	1	Male	105	47.30
	2	Female	116	52.25
		Missing	1	0.45
Gender combination (self-and supervising evaluators)	1	Same gender	123	55.41
	2	Different gender	97	43.69
		Missing	2	0.90
Duration of working in same section (months)	1	< 6	0	0.00
	2	≥ 6 and < 12	93	41.89
	3	≥ 12 and < 18	39	17.57
	4	≥ 18 and < 24	39	17.57
	5	≥ 24 and < 30	16	7.21
	6	≥ 30	35	15.77
Weekly working days in the same room	1	< 1	17	7.66
	2	1	10	4.50
	3	2	8	3.60
	4	3	25	11.26
	5	4	56	25.23
	6	≥ 5	106	47.75
Responsible for training self-evaluator	1	Yes	124	55.86
	2	No	98	44.14
Evaluation time (mins)	1	< 15	163	73.42
	2	15–29	52	23.42
	3	30–44	4	1.80
	4	≥ 45	3	1.35

This includes all MLSs in the department, encompassing part-time, temporary and non-regular staff

Most self-evaluators had 6 to < 12 months of experience (*n* = 45, 31.3%), followed by 18 to < 24 months (*n* = 40, 27.8%) and 30 to < 36 months (*n* = 39, 27.1%). Fewer had 12 to < 18 months (*n* = 13, 9.0%) or 24 to < 30 months (*n* = 7, 4.9%), with none having less than 6 months.

Among 222 supervising evaluators, experience was distributed as follows: 10–19 years (*n* = 115, 51.8%), 20–29 years (*n* = 62, 27.9%) and 30 or more years (*n* = 45, 20.3%). Most worked with self-evaluators more than 5 days per week (*n* = 106, 47.8%), followed by 4 days (*n* = 56, 25.2%), 3 days (*n* = 25, 11.3%). The duration in the same section as the self-evaluator was primarily 6 to < 12 months (*n* = 93, 41.9%), followed by 12 to < 18 months (*n* = 39, 17.6%), 18 to < 24 months (*n* = 39, 17.6%), 24 to < 30 months (*n* = 16, 7.2%) and 30 or more months (*n* = 35, 15.8%). None had less than 6 months in the same section, consistent with the inclusion criteria.

All self-evaluators (*n* = 144, 100.0%) and most supervising evaluators (*n* = 215, 96.8%) completed evaluations within 30 min, with 84.7% of self-evaluators and 73.4% of supervising evaluators finishing within 15 min.

No notable differences in demographic characteristics were observed between the ‘All Participants’ and ‘Final Samples’.

### Item analysis

The final items for analysis, prior to EFA, are presented in Fig. 2, showing no ceiling or floor effects. Item-total correlation coefficients ranged from 0.42 to 0.74. Good–Poor analysis revealed statistically significant differences (*p* < 0.001) between the top and bottom quartiles for all items.

### Construct Validity Analysis (Structural Validity)

#### EFA

The KMO was 0.91, and Bartlett’s test of sphericity was significant, χ^2^ (820) = 4018.04, *p* < 0.001. The EFA was performed on the self-evaluation dataset using maximum likelihood estimation with Promax rotation. The fourth factor’s eigenvalue was 1.025, and a four-factor solution was supported by the scree plot and theoretical considerations. One item was excluded due to a factor loading below 0.40. The final solution comprised four factors and 40 items, all with loadings exceeding 0.40. Factor titles were determined through thorough discussions, reviewing all items within each factor. The factors were designated by the following names: (1) Basic Requirements for Healthcare Professionals, (2) Laboratory Practices, (3) Additional Essential Practices and (4) Self-Development and Continuing Education. The cumulative variance explained by the four factors was 52%, with inter-factor correlations ranging from 0.46 to 0.64. Results are presented in Table 2.

**Table 2:** Items and EFA factor loadings

Factors and Items		Factor Loadings
**Factor 1. Basic Requirement for Healthcare Professionals**		
B1	Understands the safe and appropriate environment of the laboratory	0.44
B2	Identifies unsafe work practices and regulatory breaches and consults if necessary	0.45
B3	Ensures safe specimen transport and disposal according to established protocols based on knowledge of biohazard differences	0.41
B4	Understands infection prevention measures and implements them in practice	0.53
B5	Ensures timely reporting, clear communication and appropriate consultation with colleagues and supervisors	0.73
B6	Interacts respectfully with patients (examinees)	0.81
B7	Communicates appropriately with healthcare professionals and other relevant personnel, including clinical trainees	0.83
B8	Under supervision and assistance, fulfils the responsibilities of a laboratory team member	0.55
B9	Under direct supervision, understands the roles of other healthcare professionals and collaborates with them	0.60
B10	Reports actions or inactions (omissions) and implements measures to prevent recurrence	0.55
B11	Expresses personal opinions appropriately according to the situation	0.50
B12	Maintains awareness as a member of the organisation and performs assigned tasks responsibly	0.78
B13	Understands the principles of the medical institution and the goals of the laboratory	0.58
B14	Demonstrates ethical behaviour as a healthcare professional	0.86
B15	Perform tests without compromising patient (examinee) safety	0.50
**Factor 2. Laboratory Practices**		
L1	Performs specimen reception appropriately in accordance with standard protocols	0.67
L2	Prepares all necessary equipment and supplies for analyses	0.62
L3	Prioritises analyses based on urgency and workflow efficiency	0.66
L4	Performs analyses accurately, adhering to proper procedures and techniques	0.68
L5	Reports test results based on appropriate internal quality control protocols	0.80
L6	Assesses the validity of test results based on clinical information and previous results	0.59
L7	Considers repeat or additional testing as necessary	0.50
L8	Interprets verified results and reports them based on supporting evidence	0.59
L9	Under direct supervision, effectively communicates critical and alert values to the care team	0.71
L10	Uses the laboratory information system appropriately for retesting and reporting test results	0.66
L11	Stores and disposes of test results and reports in compliance with personal information protection policies	0.53
L12	Understands manuals and protocols accurately	0.52
L13	Performs internal quality control of the assigned testing equipment	0.60
L14	Maintains the assigned testing equipment	0.55
L15	Recognises and reports abnormalities in assigned laboratory equipment	0.50
L16	Under direct supervision, adjusts stock of laboratory supplies and testing-related materials as needed	0.41
**Factor 3. Additional Essential Practices**		
A1	Under direct supervision, interprets external quality control reports based on appropriate knowledge	0.49
A2	Understands emergency response procedures for disasters, power outages and other critical events	0.69
A3	Acts appropriately in response to patient (examinee) deterioration in accordance with hospital emergency protocols	0.49
A4	Understands basic healthcare laws and the medical insurance system (medical service fee system)	0.64
**Factor 4. Self-Development and Continuing Education**		
S1	Under direct supervision, proactively acquires professional knowledge and skills related to assigned duties, and collects necessary evidence	0.67
S2	Engages in workshops and conferences to maintain and update scientific knowledge and skills	0.72
S3	Receives the supervisors’ advice positively, identifies personal challenges, and sets individual goals	0.68
S4	Demonstrates a continuous desire for improvement, learns from mistakes and applies lessons to future development	0.87
S5	Recognises the importance of lifelong self-directed learning as a healthcare professional	0.97

#### CFA

**Fig. 3:**
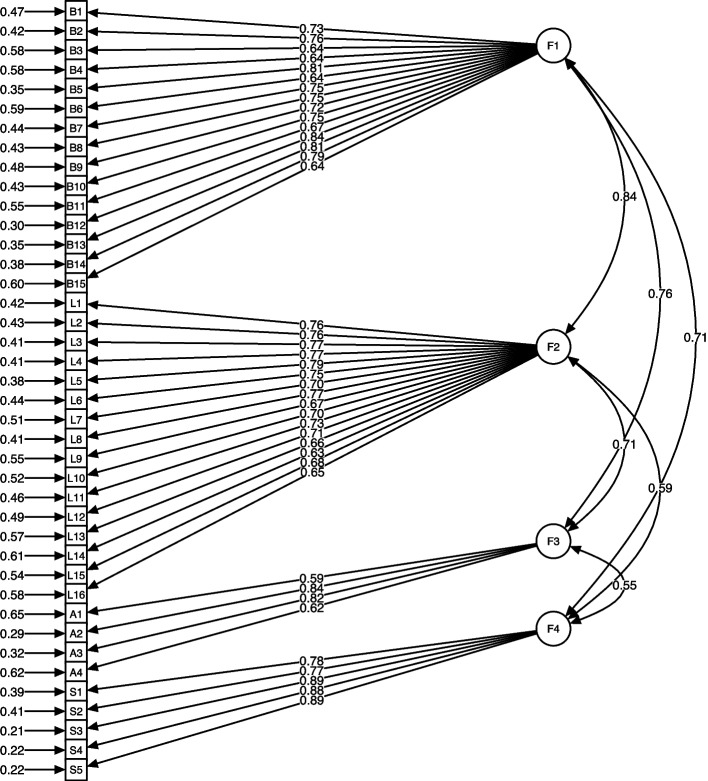
Confirmatory factor analysis model of the J-MLSCS. *Note:* Circles represent latent variables (factors), and squares represent observed variables (items). Single-headed arrows denote standardised factor loadings, and double-headed arrows indicate correlations between factors. Numbers to the left of squares represent error variances of the observed variables. F1 = Basic Requirement for Healthcare Professional, F2 = Laboratory Practice, F3 = Additional Essential Practice, F4 = Self-Development and Continuing Education

The model showed CFI = 0.814, TLI = 0.803, RMSEA = 0.088 and SRMR = 0.064 (Table 3). The resulting scale, comprising four factors and 40 items, was designated the Japan Medical Laboratory Scientists Competency Scale (J-MLSCS). The structural model and standardised path coefficients are shown in Figure 3. Additional details on the J-MLSCS are provided in Additional file 1.

**Table 3:** Summary of factor analyses

Analysis Type	Dataset Used	Sample Size	Method	Factor Structure and Model Fit Results
EFA	Self-evaluation samples	*N* = 144	ML, Promax rotation	KMO = 0.91, 4-factor model
CFA	Supervisory evaluation samples	*N* = 222	ML, NLMINB	CFI = 0.814, TLI = 0.803 RMSEA = 0.088, SRMR = 0.064

### Reliability Analysis

Internal consistency analysis yielded a Cronbach’s alpha of 0.96 for the overall scale, with factor-specific values ranging from 0.78 to 0.94.

### Scores by self-evaluators’ experience levels

**Fig. 4:**
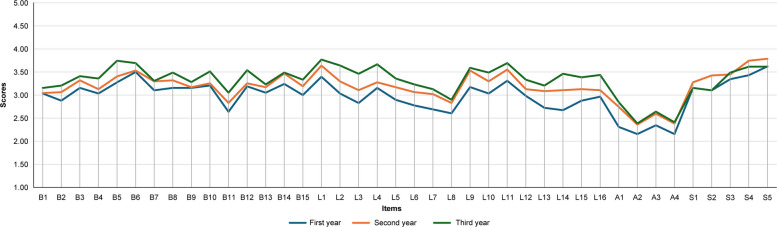
Self-Evaluation scores by experience level. Note: Self-evaluators were grouped by Experience: < 1 year (first-year), 1 to < 2 years (second-year) and 2 to < 3 years (third-year)

**Fig. 5:**
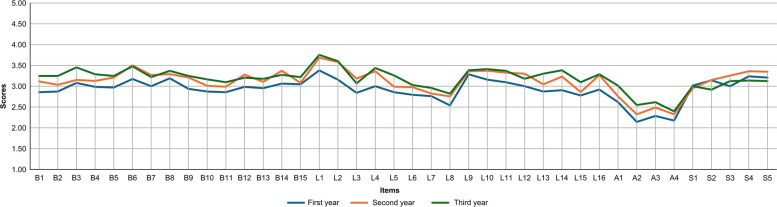
Supervisory evaluation scores by self-evaluator experience level. Note: Self-evaluators were grouped by experience: < 1 year (first-year), 1 to < 2 years (second-year) and 2 to < 3 years (third-year)

Comparisons across experience groups indicated that third-year self-evaluators generally scored higher than first-year self-evaluators in both self- and supervisory evaluations for most items. The largest difference was observed for item L14, ‘Maintains the assigned testing equipment’, with mean differences of 0.79 (*p* < 0.05) for self-evaluations and 0.48 (*p* < 0.05) for supervisory evaluations. However, the Self-Development and Continuing Education factor showed the highest scores for the second-year group (Figs. 4 and 5).

## Discussion

### Novel contributions and educational implications

Competency scale development is essential for CBE for MLSs. However, psychometrically validated and standardised tools for assessing MLS competencies are scarce in many countries. Individual institutions often create their own evaluation forms, which vary in structure, content and methodological rigour, impeding consistent competency assessment and comparability of educational outcomes across clinical training settings. To address this gap, the present study developed and validated the J-MLSCS, a comprehensive tool tailored for novice MLSs in Japan. The J-MLSCS provides a standardised framework for consistent application across clinical training institutions, enabling learners and educators to assess professional development using reliable, evidence-based criteria.

By integrating self- and supervisory assessment within a single validated framework, this study advances prior research, which typically relied on one perspective. This dual approach facilitated comparisons between perceived and observed competencies. In this study, differences between self- and supervisory assessments were particularly pronounced in Factor 1 (‘Self-Development and Continuing Education’), although these differences diminished when stratified by years of experience. This factor encompasses self-reflection, independent learning, proactive skill acquisition, feedback integration, goal-setting and continuous professional growth, behaviours often difficult to observe directly [49, 50]. These discrepancies likely arise from differing evaluative perspectives: self-assessments are more susceptible to psychological influences, such as self-efficacy, whereas supervisory assessments focus primarily on observable behaviours and technical performance [51, 52]. Educationally, such differences can be used constructively for feedback and learning. When self-assessment scores are lower than those of supervisory assessments, it may be beneficial to clarify learners’ strengths and encourage them to enhance self-efficacy. Conversely, when self-assessment scores exceed supervisory assessments, it is important to promote reflective practice based on objective behavioural evidence and to foster a more realistic self-perception.

Overall, the J-MLSCS was developed and validated in this study as a tool for both self- and supervisory assessments. By employing the same instrument for subjective and objective evaluation, the scale provides a versatile tool for assessing the competencies of novice MLSs across diverse educational and clinical settings in Japan. In future practice, the J-MLSCS could serve as a formative assessment tool in clinical laboratory training. Longitudinal research is needed to examine its impact on professional development, self-directed learning and supervisor-learner communication over time.

### Key results and interpretation

The J-MLSCS was validated as a reliable instrument for assessing competencies from both self- and supervising evaluator perspectives.

Item analysis and EFA on the self-evaluator dataset confirmed satisfactory psychometric properties, with adequate discrimination and internal consistency, as evidenced by item-total correlations and Good–Poor analyses. The EFA produced a coherent four-factor structure, with inter-factor correlations supporting Promax rotation to account for related but distinct constructs. Cronbach’s alpha coefficients indicated strong internal consistency across all factors. The CFA on the supervisory evaluation dataset met the minimum thresholds for acceptable fit, although it indicated room for improvement in future refinements. Moderately high correlations among latent factors (0.55–0.84), suggesting partial construct overlap, combined with the sample size, likely contributed to the relatively low CFI and TLI values. Future studies should employ larger samples and further item refinement to improve model fit and strengthen structural validity.

In this study, all supervising evaluators had worked with their corresponding self-evaluators in the same section for at least 6 months, ensuring sufficient observation time. This methodological feature enhances the reliability of supervisory ratings by minimising bias from limited acquaintance, a common issue in peer- or supervisor-based evaluations.

### Structure of the four-factor J-MLSCS and theoretical justification

**Fig. 6:**
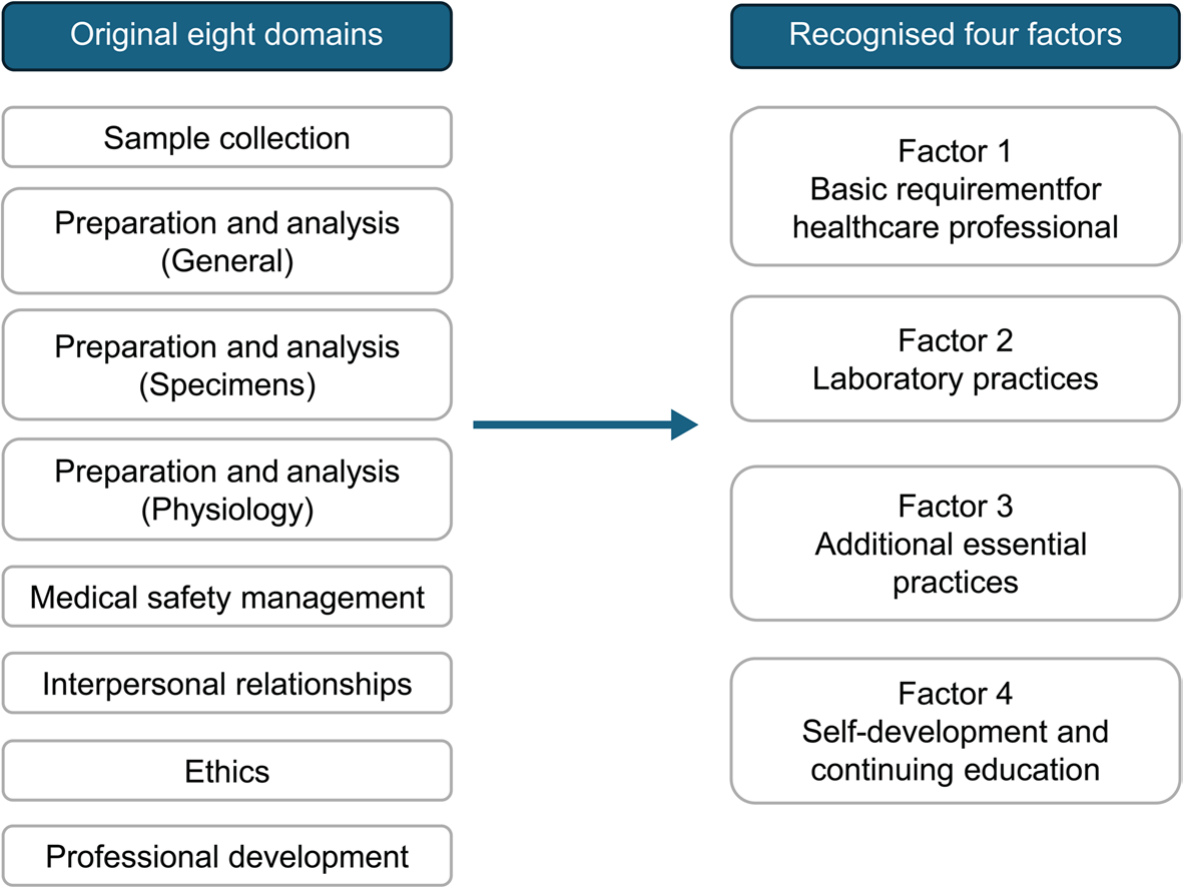
The original eight domains and recognised four factors

The four-factor structure can be conceptually explained in relation to the original eight domains established through the Delphi process (Fig. 6). Three domains—Sample Collection, Preparation and Analysis (Specimens) and Preparation and Analysis (Physiology)—exhibited high frequencies of N/A responses and were excluded from factor analysis. This exclusion significantly influenced the reorganisation into a four-factor structure. Conceptually, the extracted factors closely correspond to the remaining original domains. Factor 1 (‘Basic Requirements for Healthcare Professionals’) items from ‘Medical Safety Management’, ‘Interpersonal Relationships’ and ‘Ethics’. Factor 2 (‘Laboratory Practices’) is consistent with Preparation and Analysis (General). Factor 4 (‘Self-Development and Continuing Education’) aligns directly with the original Professional Development domain. The most substantial change appears in Factor 3 (‘Additional Essential Practices’), which integrates items from multiple original domains representing less routinely encountered but essential tasks, such as emergency response.

In this study, competencies for novice MLSs were conceptually organised into four aggregated groups, extending beyond individual test-specific skills to include fundamental abilities expected of novice MLSs as healthcare professionals. Items in Factor 3, in particular, require dedicated practical experience and guided instruction. Theoretically, this four-factor structure offers a coherent and justifiable framework for core competencies among novice MLSs.

### Experience-related trends in competency acquisition

Analysis of score distributions by self-evaluators’ experience (first-, second- and third-year) revealed no ceiling or floor effects in either self- or supervisory ratings. The scale thus appropriately captures variability in competencies at this career stage. A score of 3 (‘Acceptable’) was set as the target by the end of the third year, while 5 (‘Outstanding’) allows evaluation of advanced competencies. These findings indicate sufficient sensitivity for evaluating novice MLSs through their third year. Although developed for novices, the scale may require modifications for more experienced MLSs; however, inclusion of options for higher competency levels permits broader application beyond novices alone.

In addition, for most items, third-year ratings were higher than first-year ratings in both self- and supervisory assessments, indicating that the J-MLSCS effectively captures the progression of skill acquisition in early clinical practice across both assessment types. Further, participants in the second-year MLSs achieved the highest scores in both self- and supervisory evaluations, suggesting proficiency in routine tasks alongside active engagement in professional development. Conversely, third-year MLSs scored lower, potentially due to increased workload and expectations limiting time for self-development, with supervisory evaluations possibly reflecting heightened evaluator expectations.

### Limitations and future research directions

This study has limitations that suggest directions for future research. First, the sample included hospitals of varying sizes, based on bed count, laboratory scales and MLS numbers, indicating a fair representation of the target population. Although all eligible hospitals nationwide meeting the inclusion criteria were invited, the final sample size was limited, potentially affecting the generalisability of the findings. Additionally, self- and supervisory assessments were collected using linked identifiers within the same hospitals to facilitate paired analyses. Although essential for the study design, this approach may have introduced correlated error and shared-method variance. Those should be considered when interpreting results. Future studies with larger, more diverse samples could further validate the J-MLSCS and enhance its applicability across clinical settings. Larger samples may also allow analysis of additional competency items, enabling a more comprehensive evaluation scale that reflects the full scope of MLS practice in Japan.

Second, several items were excluded from analysis due to a high frequency of N/A responses, likely reflecting the diverse scope of practice among Japanese MLSs, who perform laboratory testing, specimen collection and physiological examinations. MLSs rotate through different laboratory sections to fulfil these roles; however, these rotation systems are not standardised nationwide and are determined independently by each institution. Importantly, N/A responses helped distinguish between inapplicability due to task rotation and gaps in exposure or training. This pattern indicated that N/A selections were typically consistent across all items in a domain when related to rotation systems, whereas they appeared more sporadically when reflecting limited practical experience. Consequently, some participants had not engaged in certain tasks at the time of evaluation, and recruiting evaluators with comprehensive knowledge of all practice areas was challenging due to the limited number of eligible MLSs. Despite these challenges, the finalised J-MLSCS was carefully designed to align with the actual task rotation practices in Japan, incorporating items that reflect the patient-facing tasks of novice MLSs. This enhances its relevance and feasibility as a context-sensitive evaluation tool. Although some items were excluded from statistical analysis, their content validity was confirmed through the Delphi method, preserving their utility for educational and training purposes. Future iterations could consider task-specific subscales or modular applications of the tool.

Finally, this study did not assess test–retest reliability, but the J-MLSCS holds potential for longitudinal assessments to track novice MLSs’ developmental trajectories. Future longitudinal or practice-based research could confirm the scale’s temporal stability and provide insights into how MLS competencies evolve in clinical settings. Additionally, while the scale was developed in Japanese and translated into English, the psychometric properties of the English version remain unvalidated. These findings are context-specific to novice MLSs in Japan; cross-cultural validation is required before applying the J-MLSCS in other countries or healthcare systems.

### Practical applications of the J-MLSCS

The J-MLSCS, reflecting the unique roles and responsibilities of Japanese MLSs, provides a robust foundation for structured training and competency-based evaluation in clinical laboratories. Despite limitations in sample representativeness and generalizability, this study marks a significant step towards evidence-based human resource development in the field.

Beyond individual competency assessment, the J-MLSCS has the potential to inform the design and improvement of educational programmes and curricula. Its systematic use can enable comparisons across institutions, facility sizes and time points. Furthermore, adapting the scale for pre-graduate education and clinical training programmes could strengthen the professional development pipeline for MLSs in Japan. Its effectiveness would be maximised in settings implementing CBE.

## Conclusion

This study developed and validated the J-MLSCS, a 40-item competency assessment scale across four factors tailored for novice MLSs in Japan. The scale demonstrated robust reliability and validity, supporting its use as a practical tool for self- and supervisory evaluations. Comparing self- and supervisory assessments can identify gaps between subjective and objective evaluations, informing targeted educational interventions and fostering professional development for MLSs.

## Supplementary Information

Supplementary Material 1.

## Data Availability

The datasets used and/or analysed during the current study are available from the corresponding author upon reasonable request.

## Abbreviations

CBE: Competency-based education
ACGME: Accreditation Council for Graduate Medical Education
MLS: (Bio) Medical Laboratory Scientist
IFBLS: International Federation of Biomedical Laboratory Science
CDC: Centres for Disease Control and Prevention
ASCP: American Society for Clinical Pathology
COSMIN: COnsensus-based Standards for the selection of health Measurement INstruments
SD: Standard deviation
N/A: Not applicable
EFA: Exploratory factor analysis
ML: Maximum likelihood
KMO: Kaiser–Meyer–Olkin
CFA: Confirmatory factor analysis
J-MLSCS: Japan Medical Laboratory Scientists Competency Scale
