# Supplementary material for: Development and validation of a dual-perspective competency scale for novice medical laboratory scientists in Japan
Source: BMC Med Educ. 2026 Jan 19;26:544. doi: 10.1186/s12909-026-08626-9 (PMC13041148; doi:10.1186/s12909-026-08626-9)
Supplement: Supplementary file 1 — Supplementary Material 1. [file 12909_2026_8626_MOESM1_ESM.pdf]

Development and Validation of a Dual-perspective Competency Scale for Novice Medical Laboratory Scientists in Japan

Kiriko Maekawa

Supplementary material 1:

The Japan Medical Laboratory Scientists Competency Scale: J-MLSCS

Purpose	To measure the (Bio) Medical Laboratory Scientist Competency
Methods	The Competency of Japanese Novice Medical laboratory scientists (Maekawa, 2022) was used as a research framework, and the developed as a scale.
Type	Self-evaluation<br>5-points Likert scale (1 = Poor, 2 = Needs Improvement, 3 = Acceptable, 4 = More than Acceptable, 5 = Outstanding), with an additional option of 'Not Applicable.'<br><br>Supervising evaluation<br>5-points Likert scale (1 = Poor, 2 = Needs Improvement, 3 = Acceptable, 4 = More than Acceptable, 5 = Outstanding), with an additional option of 'Not Applicable (N/A).'
Target	Japanese Novice (Bio) Medical Laboratory Scientist Competency
Composition	Total 40 items<br><br>Basic Requirement for Healthcare Professional (15 items)<br>Laboratory Practice (16 items)<br>Additional Essential Practice (4 items)<br>Self-Development and Continuing Education (5 items)
Interpretation	Higher scores indicate higher (Bio) Medical Laboratory Scientist competency.

This document is confidential and may be used only for non-commercial educational purposes or for research purposes upon prior approval from the authors. Any research use requires contacting the authors in advance and submitting the designated application form. When using this document for such purposes, you must cite the relevant publication information.
The content of this document must not be used for commercial or for-profit activities without explicit licensing permission.

The following items examine the (Bio) Medical Laboratory Scientist competency. Read the items and mark '✓' at the ones that you think best represent your thoughts or actions.

Item Statements		Competency scale					
		1<br>Poor	2<br>Needs Improvem ent	3<br>Ac-ceptable	4<br>More than Ac-ceptable	5<br>Out-standing	Not Applicable (N/A)
**1. Basic Requirement for Healthcare Professional**							
B1	Understands the safe and appropriate environment of the laboratory.	❑	❑	❑	❑	❑	❑
B2	Recognises hazardous work practices and rule violations and consults supervisors and senior staff when necessary.	❑	❑	❑	❑	❑	❑
B3	Recognises different biohazards and safely performs specimen transport and disposal according to established protocols.	❑	❑	❑	❑	❑	❑
B4	Possesses accurate knowledge of infection prevention measures and implements them in practice.	❑	❑	❑	❑	❑	❑
B5	Effectively reports, communicates, and consults with colleagues and supervisors on work-related matters as appropriate.	❑	❑	❑	❑	❑	❑
B6	Treats patients (examinees) with respect.	❑	❑	❑	❑	❑	❑
B7	Communicates appropriately with healthcare professionals and other relevant personnel, including clinical trainees.	❑	❑	❑	❑	❑	❑
B8	With direct supervision, fulfils the responsibilities of a laboratory member.	❑	❑	❑	❑	❑	❑
B9	Under direct supervision, understands the roles of each healthcare professional and works collaboratively with them.	❑	❑	❑	❑	❑	❑
B10	Acknowledges and reports errors or oversights and implements actions to prevent recurrence.	❑	❑	❑	❑	❑	❑
B11	Communicates professional opinions appropriately according to the situation.	❑	❑	❑	❑	❑	❑
B12	Maintains awareness as a member of the organisation and performs assigned tasks responsibly.	❑	❑	❑	❑	❑	❑
B13	Understands the principles of the medical institution and the goals of the laboratory in which they work.	❑	❑	❑	❑	❑	❑
B14	Demonstrates ethical behaviour as a healthcare professional.	❑	❑	❑	❑	❑	❑
B15	Performs analyses without causing harm to the patient (examinee).	❑	❑	❑	❑	❑	❑
**2. Laboratory Practices**							
L1	Receives specimens in accordance with standard protocols.	❑	❑	❑	❑	❑	❑
L2	Prepares all necessary equipment and supplies for analyses.	❑	❑	❑	❑	❑	❑
L3	Prioritises analyses based on urgency and workflow efficiency.	❑	❑	❑	❑	❑	❑
L4	Performs analyses accurately, adhering to proper procedures and techniques.	❑	❑	❑	❑	❑	❑
L5	Verifies that test results are supported by appropriate internal quality control measures.	❑	❑	❑	❑	❑	❑
L6	Assesses the validity of test results based on the clinical patient information and prior results.	❑	❑	❑	❑	❑	❑

L7	Considers repeat or additional testing as necessary.	❑	❑	❑	❑	❑	❑
L8	Interprets verified results and reports them based on supporting evidence.	❑	❑	❑	❑	❑	❑
L9	With direct supervision, reports critical test results in a manner that highlights their importance.	❑	❑	❑	❑	❑	❑
L10	Uses laboratory system appropriately for retesting and reporting test results.	❑	❑	❑	❑	❑	❑
L11	Stores and disposes of the test results and reports in compliance with personal information protection policies.	❑	❑	❑	❑	❑	❑
L12	Understands manuals and protocols correctly.	❑	❑	❑	❑	❑	❑
L13	Manages the internal quality control of the test equipment in use.	❑	❑	❑	❑	❑	❑
L14	Maintains the test equipment in use.	❑	❑	❑	❑	❑	❑
L15	Recognises and reports abnormalities in assigned laboratory equipment.	❑	❑	❑	❑	❑	❑
L16	With direct supervision, adjusts the stock of laboratory supplies and testing-related materials as needed.	❑	❑	❑	❑	❑	❑
**3. Additional Essential Practices**							
A1	With direct supervision, understands the assessment results based on the accurate interpretations of external quality control.	❑	❑	❑	❑	❑	❑
A2	Understands appropriate actions during emergencies, such as disasters and power outages, and prepares in advance.	❑	❑	❑	❑	❑	❑
A3	Adheres to the hospital's emergency response protocols when a patient (examinee) experiences acute deterioration.	❑	❑	❑	❑	❑	❑
A4	Understands basic healthcare laws and the medical insurance system.	❑	❑	❑	❑	❑	❑
**4. Self-Development and Continuing Education**							
S1	With direct supervision, proactively acquires professional knowledge and skills necessary for assigned duties and collects required evidence.	❑	❑	❑	❑	❑	❑
S2	Engages in workshops and conferences to maintain and enhance scientific knowledge and skills.	❑	❑	❑	❑	❑	❑
S3	With direct supervision, openly receives supervisors' feedback, identifies personal challenges, and sets individual goals.	❑	❑	❑	❑	❑	❑
S4	Maintains a constant desire for improvement, learns from failures, and applies lessons to future growth.	❑	❑	❑	❑	❑	❑
S5	Recognises the importance of lifelong self-directed learning as a healthcare professional.	❑	❑	❑	❑	❑	❑

Note: These competencies are defined as the minimum requirements for novice MLSs with less than 3 years of clinical experience. It is generally assumed that novices perform their duties under supervision; however, items marked as 'with direct supervision' were suggested in the Delphi survey to be challenging for novices to perform independently.
